# RETRACTED: Saied et al. Mycosynthesis of Hematite (α-Fe_2_O_3_) Nanoparticles Using *Aspergillus niger* and Their Antimicrobial and Photocatalytic Activities. *Bioengineering* 2022, *9*, 397

**DOI:** 10.3390/bioengineering12020100

**Published:** 2025-01-22

**Authors:** Ebrahim Saied, Salem S. Salem, Abdulaziz A. Al-Askar, Fathy M. Elkady, Amr A. Arishi, Amr H. Hashem

**Affiliations:** 1 Botany and Microbiology Department, Faculty of Science, Al-Azhar University, Nasr City 11884, Egypt; 2 Department of Botany and Microbiology, Faculty of Science, King Saud University, Riyadh 12372, Saudi Arabia; 3 Microbiology and Immunology Department, Faculty of Pharmacy (Boys), Al-Azhar University, Nasr City 11884, Egypt; 4 School of Molecular Sciences, The University of Western Australia, Perth, WA 6009, Australia

The *Bioengineering* Editorial Office retracts the article “Mycosynthesis of Hematite (α-Fe_2_O_3_) Nanoparticles Using *Aspergillus niger* and Their Antimicrobial and Photocatalytic Activities” [1] cited above.

Following publication, concerns were brought to the attention of the publisher regarding particle duplication within Figure 5A in this article [1].

Adhering to our complaints procedure, an investigation was conducted by the Editorial Office and the Editorial Board. While the authors cooperated with the Editorial Office during the investigation, they were unable to satisfactorily explain the particle duplication within Figure 5A, nor could the Editorial Board validate this image from the raw material provided. As a result, the Editorial Board were unable to confirm the reliability of the findings and subsequently decided to retract this article [1] as per MDPI’s retraction policy (https://www.mdpi.com/ethics#_bookmark30, accessed on 15 January 2025) and in line with the Committee on Publication Ethics retraction guidelines (https://publicationethics.org/retraction-guidelines, accessed on 15 January 2025).

This retraction was approved by the Editor-in-Chief of the journal *Bioengineering*.

The authors did not agree to this retraction.
